# The Mesoaccumbens Pathway: A Retrograde Labeling and Single-Cell Axon Tracing Analysis in the Mouse

**DOI:** 10.3389/fnana.2017.00025

**Published:** 2017-03-27

**Authors:** Claudia Rodríguez-López, Francisco Clascá, Lucía Prensa

**Affiliations:** Departamento de Anatomía, Histología y Neurociencia, Facultad de Medicina, Universidad Autónoma de MadridMadrid, Spain

**Keywords:** dopamine, accumbens core, accumbens shell, axonal branching, parabraquial pigmented nucleus, paranigral, ventral tegmental area, tyrosine hydroxylase

## Abstract

Neurons in the ventral tegmental area (VTA) that innervate the nucleus accumbens (Acb) constitute the so-called mesoaccumbens system. Increased activity by these neurons is correlated with the expectation and achievement of reward. The mesoaccumbens projection neurons are regarded as a central node in the brain networks that regulate drive and hedonic experience, and their dysregulation is a common pathophysiological step in addictive behaviors as well as major depression. Despite previous anatomical studies that have analyzed the origin of the mesoaccumbens axons within the VTA, regarded as a unit, the exact contributions of the various cytoarchitectural subdivisions of the VTA to this innervation is still unexplored; understanding these contributions would help further our understanding of their precise anatomical organization. With the aim of deciphering the contribution of the various VTA subdivisions to accumbal innervation, the present study has used retrograde tracer microinjections in the Acb to map the location within the various VTA subdivisions of neurons targeting either the shell or core compartments of the Acb in mice. Furthermore, the dopaminergic nature of these projections has also been analyzed using tyrosine-hydroxylase immunohistochemistry. We demonstrate here that small territories of the Acb core and shell are innervated simultaneously by many VTA subdivisions, contributing dopaminergic as well as non-dopaminergic axons to the accumbal innervation. In fact, single VTA subdivisions harbor both dopaminergic and non-dopaminergic neurons that project to the same accumbal territory. The most medial VTA subnuclei, like the caudal linear nucleus, project abundantly to medial aspects of the Acb core, whereas more lateral territories of the Acb are preferentially targeted by neurons located in the parabrachial pigmented and paranigral nuclei. Overall, about half of the mesoaccumbens neurons are putatively dopaminergic in mice. Anterograde single-cell labeling (Sindbis-pal-eGFP vector) of a limited sample of neurons revealed that mesoaccumbens neurons form profuse terminal arborizations to cover large volumes of either the Acb core or shell, and, unlike other VTA projection neuron populations, they do not branch to other striatal or extrastriatal structures. These anatomical observations are consistent with reports of an intense response in many Acb neurons after stimulation of very few VTA cells.

## Introduction

The Acb is the largest component of the ventral striatum and is comprised of a dorsal core (AcbC) adjacent to the ventral CPu and a surrounding ventral shell (AcbSh) (Zaborszky et al., 1985; Voorn et al., 1989; Zahm and Brog, 1992). The two accumbal subdivisions show very similar cytoarchitectonic features in Nissl-stained material, the core having a slightly lower neuronal density (154 neurons/mm^3^ × 10^3^) than the adjacent shell (220 neurons/mm^3^ × 10^3^) in the rat (Meitzen et al., 2011). The boundary between the AcbC and AcbSh in rodents is subtle but can be visualized using opiate receptor or substance P immunoreactivities (Heimer et al., 1997). Previous studies have established that the afferent and efferent connections are segregated in the accumbal core and shell of the rat (Phillipson and Griffiths, 1985; Heimer et al., 1991; Brog et al., 1993; Wright and Groenewegen, 1995; Wright et al., 1996; Groenewegen et al., 1999; Vertes, 2004). Bulk injections of anterograde tracers performed in rats have shown that the AcbC projects primarily to the dorsal portion of the VP, the medial globus pallidus, and SNC within the dopaminergic ventral mesencephalon (Nauta et al., 1978; Heimer et al., 1991; Usuda et al., 1998), whereas the AcbSh instead projects preferentially to the medial VP, LH, VTA, SNR, and extended amygdala, in both rats (Zahm and Heimer, 1990; Heimer et al., 1991; Usuda et al., 1998) and cats (Groenewegen and Russchen, 1984). The existence of distinct AcbC and AcbSh subcircuits has been observed at the single axon level in the rat and these pathways are interconnected by numerous collaterals in their downstream projections at the pallidal and mesencephalic levels (Tripathi et al., 2010). The AcbSh participates in conditioned aversion and enhancement to addictive drugs (Fenu et al., 2001) while the AcbC is involved in the locomotor aspect of reward learning and drug addiction (Ito et al., 2004), thus generating conditioned responses based on stimulus-outcome associations made by AcbSh neurons (Ikemoto, 2007), as seen in rats. These processes are accomplished through a connectivity loop consisting of AcbSh projections to VTA neurons that, in turn, innervate AcbC, and this subnucleus would relay information back to more lateral VTA cells, which then finally arborize in the CPu (Zahm, 2000; Voorn et al., 2004).

The VTA is a large and histologically heterogeneous ventral mesencephalic territory. Several subdivisions have been identified within the rodent VTA; they include the PBP, PN, rVTA, VTT, and three midline subnuclei: the IF, RLi and CLi (Swanson, 1982; Ikemoto, 2007; Fu et al., 2012; see Aransay et al., 2015 for further discussion). Projections from VTA to Acb compose the so-called mesoaccumbens system in which dopaminergic axons modulate prefrontal, hippocampal and amygdaloid driving inputs at Acb (Brady and O’Donnell, 2004). Glutamatergic, GABAergic and dopamine-amino acid co-releasing terminals are also part of this system in rodents (Yamaguchi et al., 2011; Gorelova et al., 2012; Taylor et al., 2014). Malfunction of the equivalent neuronal networks in humans is believed to contribute to the pathophysiology of psychiatric disorders such as addiction (Wise, 1978, 2002, 2009; Berridge and Robinson, 1998; Ranaldi, 2014), schizophrenia (Sesack and Carr, 2002; Laviolette, 2007) and major depression (Nestler and Carlezon, 2006; Friedman et al., 2009; Russo and Nestler, 2013).

The anatomy of the mesoaccumbens pathway was previously explored with classic retrograde and anterograde tract tracing methods. Most retrograde studies, however, were based on large tracer injections in the rat, and these injections involved both Acb subdivisions (Swanson, 1982; Albanese and Minciacchi, 1983; Phillipson and Griffiths, 1985; Brog et al., 1993; Hasue and Shammah-Lagnado, 2002; Yamaguchi et al., 2011). Two rat studies based on smaller retrograde tracer injections shed some light on the different VTA projections to AcbC or AcbSh (Ikemoto, 2007; Lammel et al., 2008, 2011), but did not relate their findings to the VTA subnuclei that are recognized today (see above). The few available anterograde-tracing studies were carried out with low-sensitivity methods in rats (Fallon and Moore, 1978; Beckstead et al., 1979). Recently, the use of a rabies-mediated transsynaptic tracing method in mice has made it possible to demonstrate distinct patterns of axonal arborization by the VTA neurons projecting to medial and lateral sectors of the Acb (Beier et al., 2015), but that study did not provide information on the neuronal cell types of origin in VTA subdivisions.

Here, we have mapped the origin of mesoaccumbens axons in the subdivisions currently recognized in the mouse VTA. This study is designed to offer a better knowledge of the precise anatomical organization of this system in rodents, as has been done with the nigrostriatal pathway in rats (Gerfen et al., 1987; Prensa and Parent, 2001; Cebrián and Prensa, 2010). We used microinjections of retrograde tracers in the core or shell divisions of the mouse Acb to reveal the neurons innervating these accumbal territories from each of the various VTA subdivisions. In addition, we used TH double-labeling to quantify the dopaminergic phenotype in mesoaccumbens neurons. Finally, we used the Sindbis-pal-eGFP vector (Furuta et al., 2001; Aransay et al., 2015) to visualize and reconstruct the complete axonal arborization of three mesoaccumbens neurons located in different VTA subdivisions.

## Materials and Methods

### Animals

A total of 15 adult male C57BL/6 mice weighing 22–35 g were used in the present study. Of these, 14 were used for tracer studies, and 1 for cytoarchitectonic delineation of the mouse Acb and VTA. All surgical and animal care procedures were carried out in accordance with European Community Council Directive 86/609/EEC and approved by the Institutional Bioethics Committee of the Autonomous University of Madrid.

### Surgical Procedures and Histological Processing

Anesthesia was initiated with a solution of ketamine (Imalgène 500, 100 mg/kg) plus xylazine (Rompun 2%, 4–8 mg/kg) in saline injected intraperitoneally (0.45 mL/100 g body weight), and was subsequently continued with isoflurane 1–2% in oxygen by means of a mask attached to the stereotaxic apparatus (David Kopf Instruments, Tujunga, CA, USA).

### Injection of Retrograde Tracers and Tissue Processing

In eight animals, two different retrograde fluorescent tracers were injected in AcbC or AcbSh under stereotaxic guidance following Franklin and Paxinos’ (2007) atlas. FB (Diamidino compound 253/50, excitation 365 nm, emission 420 nm, Polysciences Europe GmbH, Eppelheim, Germany) dissolved in 0.1% in 0.1 M cacodylate buffer, pH 7.4, and FG (Hydroxystilbamidine methanesulfonate H-22845, excitation 385 nm, emission 615 nm; Molecular Probes Fluorochrome LLC, Denver, CO, USA) dissolved in 0.75% in 0.1 M PB, pH 7.4, were used. One tracer was injected in one hemisphere and the other tracer in the contralateral side of the same mouse (see Supplementary Table S1 for details of the tracer deposits analyzed here).

Using glass micropipettes pulled to a tip diameter of 10–15 μm, FG was iontophoresis using a positive-pulse of continuous current (1.5–2 μA, 7 s on/off intervals) for 2–3 min (Midgard Current Source, Stoelting, Wood Dale, IL, USA). In turn, 30–60 nL of FB were pressure-injected using a Picospritzer III precision electrovalve system (Parker Hannifin, Cleveland, OH, USA).

After a 7 days survival, animals were overdosed with sodium pentobarbital (16% in saline, 0.2 mL) and perfused transcardially with saline (15 mL), followed by 4% paraformaldehyde in PB (100 mL, pH 7.4). Brains were subsequently removed from the skull, postfixed in the same fixative (24 h, 4°C) and soaked in 30% sucrose in PB (48 h, 4°C), before cutting on the sagittal plane at 60 μm on a freezing microtome (Leica SM 2400).

All the sections were first wet-mounted on gelatin-coated glass slides without coverslipping and rapidly observed under fluorescence. The VTA region was examined and imaged at 10X through a UV2A Nikon filter set on a Nikon eclipse 80i epifluorescence microscope equipped with a Nikon DMX 1200 camera. All the sections containing retrogradely labeled VTA neurons were selected and incubated overnight (free-floating, 4°C) with an anti-TH monoclonal antibody (ImmunoStar; 1:1000) in PBS containing 0.1% Triton X-100 and 2% NGS. All incubations described hereafter were followed by rinses with PBS. The sections were subsequently incubated for 2 h at room temperature with an AlexaFluor 568-conjugated anti-mouse IgG goat antibody (Molecular Probes; 1:200; excitation, 579 nm; emission, >603 nm) in PBS containing 0.1% Triton X-100 and 2% NGS. Finally, these sections were mounted onto gelatinized glass slides, coverslipped with DABCO solution (Peterson, 2004) and examined under a confocal microscope (Leica TCS SP2) sequentially applying argon (364 nm) and helium–neon (543 nm) laser lines, with dichroic mirror adjustments at 390–540 and 570–640 nm, respectively, to ensure complete channel separation in order to ascertain the presence of TH immunofluorescence in the FG- or FB-labeled cell somata.

Finally, the sections containing the injection sites were examined and imaged under fluorescence using the Nikon eclipse 80i epifluorescence microscope equipped with a Nikon DMX 1200 camera and then mounted onto gelatinized glass slides, counterstained with thionin, dehydrated through passage in ascending grades of alcohol, defatted in xylene for 30–40 min and coverslipped with DePeX (Serva, Germany).

### Injection of Sindbis-pal-eGFP Vector and Tissue Processing

In six animals, the Sindbis-pal-eGFP vector (Furuta et al., 2001) was injected bilaterally in the VTA. This recombinant viral vector is based on a replication defective Sindbis virus designed to express the GFP with a membrane-targeting palmitoylation signal of GAP-43 (palGFP) under the control of a promoter of the virus. Since palGFP is produced and distributed along the somatodendritic and axonal membrane of infected neurons, this vector labels the infected neurons in a Golgi-stain manner (Furuta et al., 2001; Matsuda et al., 2009; Fujiyama et al., 2011; Aransay et al., 2015).

Under stereotaxic guidance, a suspension of Sindbis-pal-eGFP vector + 0.5% BSA diluted in PBS to a final concentration of 0.5 × 10^7^ infective particles/μl were pressure-injected bilaterally into the VTA through a glass micropipette (tip diameter 20–40 μm) attached to a Picospritzer III. Animals were allowed to recover, and returned to the cage. 48–50 h after the injection animals were perfused as described above and their brains cut into 50 μm-thick sagittal sections on a freezing microtome (Leica SM 2400). All sections containing the VTA were first wet-mounted on glass slides and examined under the appropriate epifluorescence filter set (excitation, 450–490 nm; emission, 515–565 nm) to check for the presence of pal-eGFP-expressing cell somata. Those sections containing a labeled soma were dismounted and processed, free-floating, for TH immunofluorescence as described above. The sections were then mounted, dehydrated, defatted and coverslipped. Sections were examined with the confocal microscope (Espectral Leica TCS SP5; 10% argon laser and 52% DPSS 561nm laser, 800 gain, -0.3% offset) to determine the presence of TH-immunofluorescence in the GFP-expressing cells on 1 μm-thick confocal optical slices.

To visualize the axon in full detail, the sections were placed at room temperature for 20 min in 2% hydrogen peroxide (H_2_O_2_; 30%) to remove endogenous peroxidase activity. After two rinses in PB with 1% Triton X-100, the sections were incubated overnight at room temperature with rabbit anti-GFP antibody (Exbio; 1:500), 2% Triton X-100, 3% NGS and 1% BSA in PB. The next day the sections were rinsed several times in PB and then incubated for 2 h at room temperature with biotinylated goat anti-rabbit IgG (Chemicon; 1:100), 2% Triton X-100, 3% NGS and 1% BSA in PB. Finally, the sections were incubated overnight at 4°C under agitation in a solution containing ABC Elite (Vector, PK 6100) 1:100 in PB 0.1M plus 2% Triton X-100. After several rinses in PB the bound peroxidase was revealed using the glucose oxidase-diaminobenzidine-nickel method (Shu et al., 1988), and the sections were serially mounted onto gelatinized glass slides, counterstained with thionin, dehydrated through passage in ascending grades of alcohol, defatted in xylene for 30–40 min and coverslipped with DPX (BDH Gurr^®^).

### Data Analysis

#### Neurochemical Delineation of Acb and the VTA Subdivisions

**FIGURE 1:**
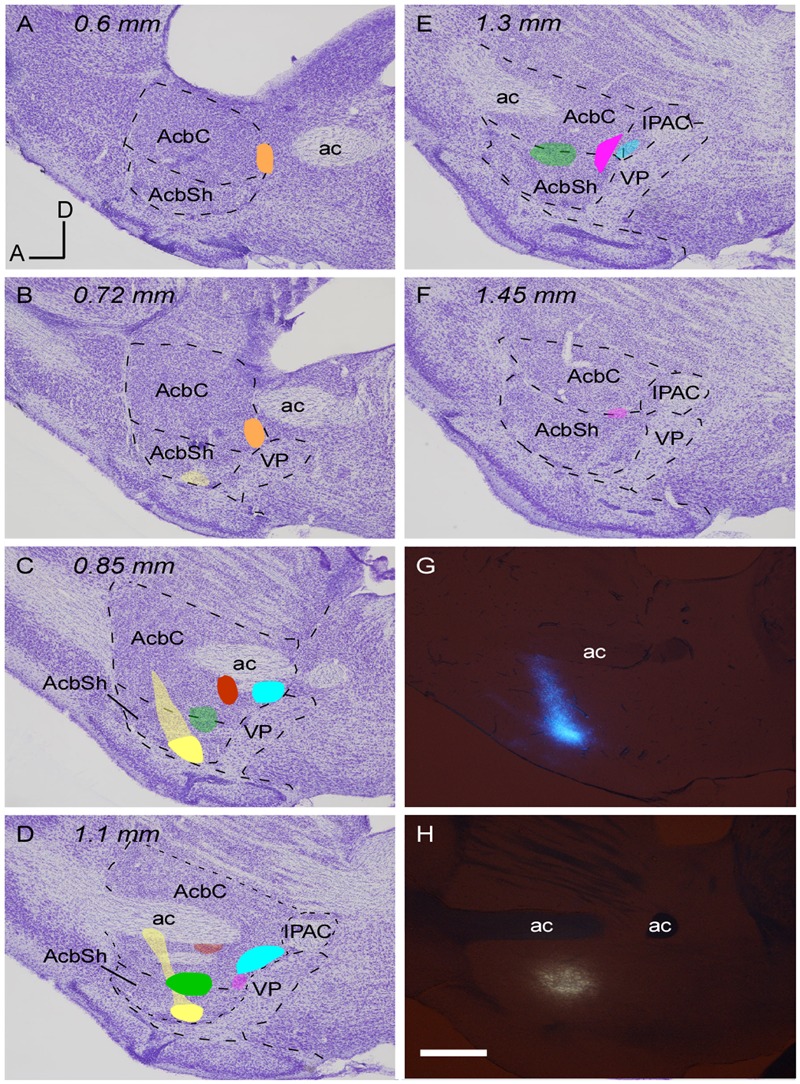
**Location of the retrograde tracer injections in the Acb. (A–F)** Sagittal sections of a mouse prosencephalon with Nissl staining showing the localization and extension of the six injections performed at Acb depicted in different colors. The densest part of each injection site corresponds to the core while the blurred zone represents the halo where little tracer was presumably taken up. Distance in millimeters to the midline plane is indicated on the top. **(G,H)** Sagittal photomicrographs of non-dehydrated sections containing the tracer deposits color coded in yellow **(G)** and green **(H)**, simultaneously taken with fluorescence and white light from the visible spectrum; this revealed the white matter borders and allowed us to locate the deposits as within or outside the Acb before proceeding to Nissl staining. Bar: 500 μm.

To delineate the Acb, a series of sagittal sections of the prosencephalon of a non-injected mouse were stained for calbindin, acetylcholinesterase and Nissl and employed to define the boundaries of the core and shell accumbal territories, as set out in Franklin and Paxinos’ (2007) atlas. The Acb extends from 0.24 to 2.15 mm from the midline in the mediolateral axis but AcbC only extends between 0.6 and 1.45 mm (**Figures 1A–F**). The rostral pole, a subregion identified by some authors (Zahm and Heimer, 1993), was not considered here as a third area because the limits between it and the core subdivision were unclear (Tripathi et al., 2010).

**FIGURE 2:**
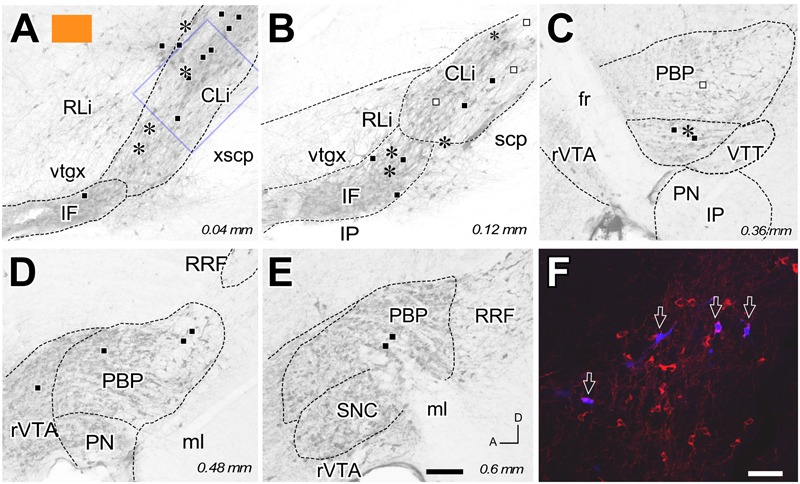
**Retrograde labeling from the FB deposit color-coded in orange in **Figure 1**. (A–E)** Distribution of the retrogradely labeled mesoaccumbens VTA neurons represented on standard planes of parasagittal VTA sections; distance in millimeters to the midline plane is indicated in the right bottom corner. Each asterisk represents a single TH+ cell and each square a TH– cell. Solid black color indicates ipsilateral neurons; open symbols indicate contralateral cells. **(F)** Confocal microscopy image corresponding to the blue square inset in **(A)**. Four FB-labeled neurons (arrows) can be seen among many other TH-immunoreactive cells (red). Bars: **A–E** = 200 μm; **F** = 50 μm.

**FIGURE 3:**
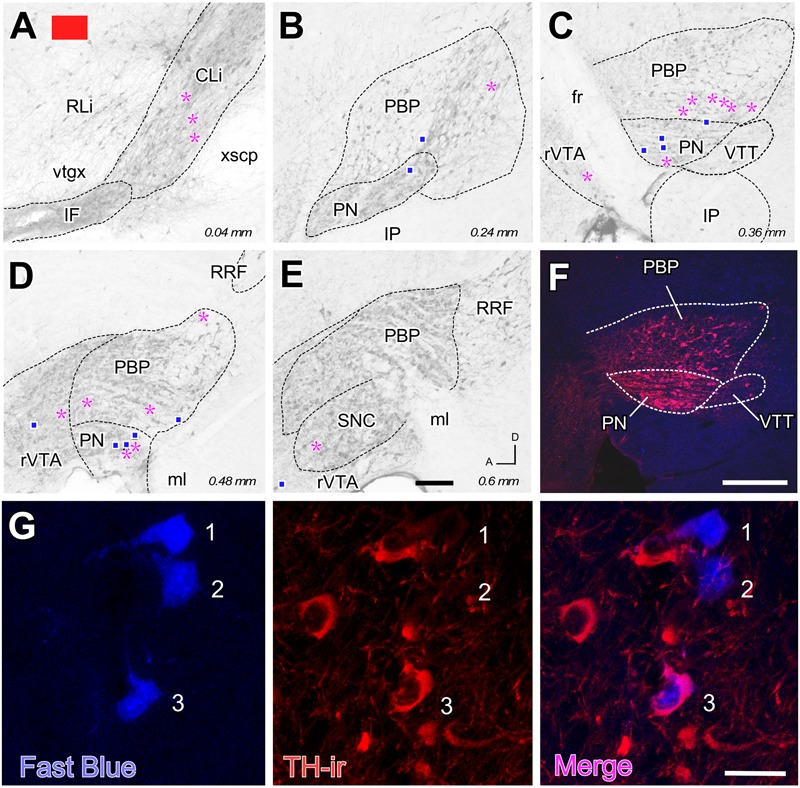
**Retrograde labeling from the FB deposit color-coded in red in **Figure 1**. (A–E)** Distribution of the retrogradely labeled mesoaccumbens VTA neurons. Each magenta asterisk represents a single TH+ cell and each blue square a TH– cell. Other conventions are as in **Figure 2**. **(F)** Conventional epifluorescence double-exposure image showing TH-immunofluorescence (red) and FB (blue). **(G)** Double-labeling confocal microscope analysis. From left to right, the same 1 μm-thick optical section is seen through excitation-emission filtering setups selecting FB or TH immunofluorescence (left and center panels). Merging both images unequivocally reveals that two of the FB-labeled cells (#1 and #2) do not show TH immunoreactivity, while a third FB-labeled cell (#3) is positive for TH. Bars: **A–E** = 200 μm; **F** = 300 μm; **G** = 30 μm.

**FIGURE 4:**
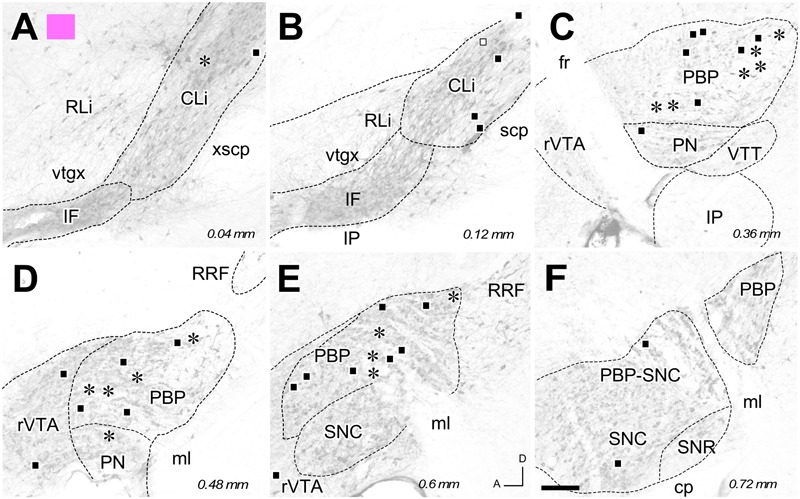
**Retrograde labeling from the FG deposit color-coded in pink in **Figure 1**. (A–F)** Distribution of the retrogradely labeled mesoaccumbens VTA neurons. Each asterisk represents a single TH+ cell and each square a TH– cell. Other conventions are as in **Figure 2**. Bar: 200 μm.

Another series of sagittal sections of the mesencephalon of the same mouse covering the SNC/VTA/RRF complex were immunostained for TH so as to define the boundaries of these structures and the four major subdivisions of VTA according to Franklin and Paxinos’ (2007) and Ikemoto (2007) atlas (i.e., PBP, PN, VTT and rVTA) (see Aransay et al., 2015 for further details and **Figures 2–4** for graphic delimitation of each subdivision); the midline nuclei IF, RLi and CLi were also defined. The protocol for TH immunohistochemistry was essentially the same as that described above except that the secondary antibody was a biotinylated horse anti-mouse IgG (Vector; 1:250). PBP stretches from 0.24 to 1.08 mm lateral to the midline and this large subdivision has been subdivided into a medial sector that stretches from 0.24 to 0.6 mm lateral to the midline, limiting ventrally with PN, and a lateral sector that occupies the region from 0.6 to 1.08 mm lateral to the midline and lies dorsal to SNC; this lateral sector also contains a caudal triangular area that is separated from the more rostral PBP by the ml. From 0.72 to 1.08 mm lateral to the midline the boundary between SNC and PBP is not clearly defined, and the neurons located at these lateral levels in the dorsal half of what is called SNC in Franklin and Paxinos’ (2007) atlas are considered part of a PBP-SNC transition area in the present work; the ventral half of SNC has been considered as the SNC proper.

#### Retrograde Labeling Analysis

The injections of the retrograde tracers resulted in a total of six deposits of tracer virtually confined to the Acb (**Figure 1** and **Table 1**). In each experiment, every single section through the region of the VTA was mounted for examination in the fluorescence microscope to determine the location and number of the retrogradely labeled cells. To ensure consistency and avoid bleaching, retrogradely labeled VTA cells were identified and plotted on the fluorescent images using Canvas X software (ACD Systems International). Only structures that were labeled with the retrograde tracers and that had clear neuronal soma shapes were considered as retrogradely labeled neurons. Other structures with faint labeling that lacked a clearly neuronal soma-like shape were discarded from the count. The parallel confocal microscopy images of the same sections were used to distinguish the FB- or FG-containing cell somata that were double-labeled by TH immunofluorescence from those that were negative for TH (TH-). To provide accurate data regarding the contribution of dopaminergic and non-dopaminergic neurons to the accumbal innervation, only clearly TH-positive (TH+) neurons filled with retrograde tracer were counted in the present work, and those retrogradely labeled neurons whose TH-staining could not be clearly assessed under confocal microscopy were considered non-dopaminergic.

**Table 1:** Percentage distribution of the retrogradely labeled neurons in each VTA subdivision.

CASE n° of neurons Tracer location	PN	medial PBP	lateral PBP	rVTA	IF	CLi	RLi	VTT	SNC
Orange *n* = 36 AcbC	8.3% (1+2)	11.1% (0+4)	5.5% (0+2)	2.8% (0+1)	16.7% (2+4)	50% (5+13)	5.5% (1+1)		
Red *n* = 31 AcbC	32.3% (3+7)	41.9% (10+3)		12.9% (2+2)		9.7% (3+0)			3.3% (1+0)
Pink *n* = 45 AcbC+AcbSh	4.4% (1+1)	44.4% (10+10)	28.9% (4+9)	6.7% (0+3)		15.5% (1+6)			
Yellow *n* = 217 AcbSh	16.1% (19+16)	32.2% (42+28)	22.6% (30+19)	7.8% (10+7)	0.5% (0+1)	7.4% (7+9)	0.5% (1+0)	0.5% (0+1)	3.2% (2+5)
Green *n* = 129 AcbC+AcbSh	7.7% (6+4)	35.7% (19+27)	34.9% (24+21)	6.9% (2+7)		6.2% (4+4)	0.8% (1+0)	0.8% (1+0)	6.9% (6+3)
Blue *n* = 38 AcbC	7.9% (2+1)	63.2% (14+10)		5.3% (0+2)		21.1% (3+5)	2.6% (0+1)		

*Absolute number of neurons is indicated between parentheses: the first digit corresponds to dopaminergic neurons and the second to non-dopaminergic neurons. The so-called PBP-SNC continuum has been included in the lateral PBP column in search of clarity.*

#### Single Neuron Tracing

The cell bodies, dendrites and entire axonal trajectories of three pal-eGFP infected VTA neurons projecting to the Acb were reconstructed using a microscope (Nikon Eclipse 50i) attached to a camera lucida employing 20x and 40x objectives. Axon drawings were digitalized with a scanner (HP Scanjet 7400c) and redrawn using Canvas X software (ACD Systems). Original TIFF images from terminal fields were processed with ImageJ software to obtain an orthogonal projection of a section, thus illustrating the visual density of each field.

## Results

### Tracer Deposits

A total of six small injections of retrograde tracers (three FB and three FG) involving the Acb were analyzed in the present study (**Figure 1** and **Table 1**). They were all located ventral to the ac, extending from 0.6 to 1.45 mm. It has to be noted that the mouse AcbC and AcbSh are rather small and slab-shaped and for that reason some of our deposits affected both Acb divisions, or encroached faintly upon adjacent structures. Three deposits (coded in orange, blue and red in **Figure 1** and **Table 1**) were restricted to the AcbC, those coded in blue and orange very slightly contaminated the adjacent BST, and the one coded in red left a small light deposit in CPu along the trajectory of the tip of the pipette. The deposit coded in orange was confined to the caudal and ventromedial corner of the AcbC (**Figures 1A–C**), the red one was located below the ac at the mid-rostrocaudal axis of the AcbC (**Figures 1C,D**), and the blue one was located at the most caudal region of the AcbC, just rostral to the boundary with VP (**Figures 1C–E**). The tracer deposit color-coded in yellow in **Figure 1** was mainly located at the mid-rostrocaudal and ventral aspect of the AcbSh (**Figures 1B–D,G**), despite a faint halo of tracer extending anterodorsally into AcbC. Two other tracer deposits, which are coded in pink and green in **Figure 1**, were restricted to the boundary between the two Acb subdivisions. Approximately three quarters of the one coded in pink was located in AcbC with the remaining quarter deposit in the dorsal aspect of AcbSh (**Figures 1D–F**). The FB injection coded in green was placed half at ventral AcbC and half at dorsal AcbSh in the central rostrocaudal third of the Acb (**Figures 1C–E,H**).

### Retrograde Labeling

The distribution of the neurons that were retrogradely labeled from each tracer deposit in the various VTA subdivisions is summarized in **Table 1**. **Table 1** also shows the number of TH+ and TH- neurons that were retrogradely labeled from each injection site. Graphic representations of the retrograde labeling provided by three of the six deposits, each covering a different mediolateral level, are depicted in **Figures 2–4** (for the other three cases see Supplementary Figure S1). Our results clearly demonstrate that small territories of the AcbC and AcbSh in mice are simultaneously innervated by many neurons from various VTA subdivisions. Furthermore, we also show that VTA neurons innervating each of these small Acb territories are neurochemically distinct, some of them being TH+ and others TH-, and that both TH+ and TH- neurons project to a given accumbal territory from a single VTA subdivision.

With respect to the overall contribution of the various VTA subdivisions to the innervation of the accumbal territories explored here, our data indicate that the PBP subdivision is the one that harbors the greatest number of retrogradely labeled mesoaccumbens neurons. Specifically, the greatest percentage of retrogradely labeled neurons is found in the medial sector of PBP, here called the medial PBP (32 to 63% of the total). An exception to this predominance of PBP retrogradely labeled neurons was observed when the caudal and ventromedial corner of the AcbC was injected (see orange deposit in **Figures 1A,B**); this particular accumbal territory was much more abundantly reached by neurons located in the CLi subdivision, these neurons constituting as much as nearly 50% of the total number of labeled neurons, more neurons than from PBP or any other VTA subdivision (**Figure 2** and **Table 1**). It is also worth noting that the CLi contributed many neurons to the innervation of two other caudal and more lateral ventral territories of the AcbC lying just rostral to the boundary with VP (see the pink and blue tracer deposits depicted in **Figure 1** and **Table 1**). However, the contribution of the CLi to the innervation of these more laterally placed ventrocaudal AcbC territories is much lower than the contribution by the PBP. According to these results, the CLi is an important source of projections to caudal sectors of the AcbC and its contribution decreases from medial to lateral AcbC territories. The contribution of the IF to the innervation of the caudal and ventromedial corner of the AcbC is also very dense, and seems to be very selective for this accumbal zone (**Figure 2** and **Table 1**). In contrast to neurons from the CLi and IF, the RLi retrogradely labeled neurons were very scarce in all cases (**Table 1**).

**FIGURE 5:**
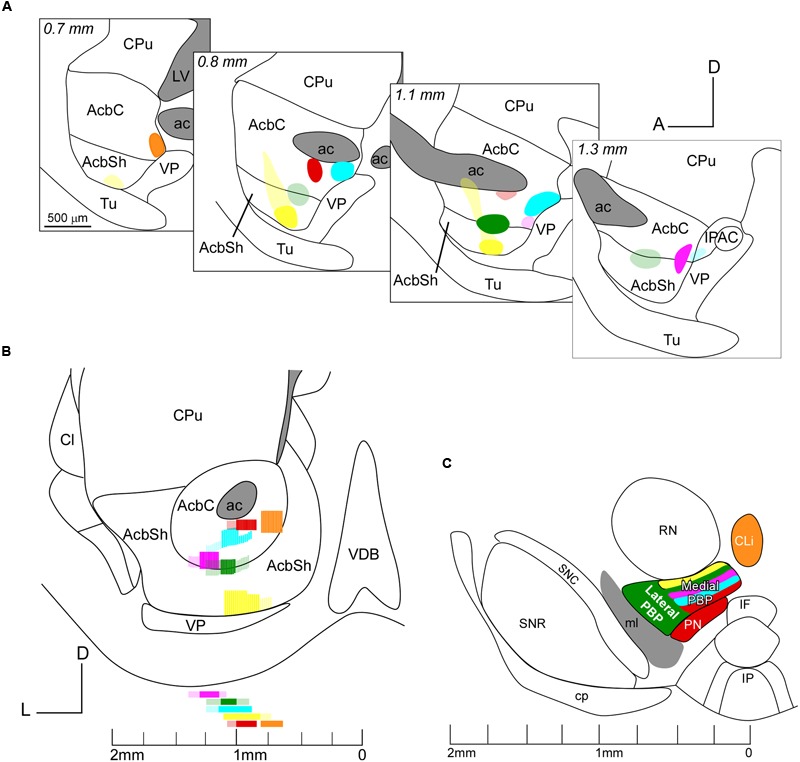
**Sagittal (A)** and coronal **(B)** views of the six deposits of the retrograde tracers at the AcbC and AcbSh. Note that the injection sites are color-coded as in **Figure 1** and that the core and peripheral halo of the deposits are, respectively, indicated by intense or light intensity. **(C)** Distribution of the mesoaccumbens pathway cells of origin in various VTA subdivisions. Only those subdivisions that contained more than 30% of the retrogradely labeled neurons per injection, according to **Table 1**, are represented in the figure. Note the intermingling of cells projecting to various sectors of the AcbC and AcbSh in the medial PBP and that neurons located more dorsally in this medial PBP innervate ventral aspects of the AcbC and AcbSh. Note also that the CLi projects intensively to caudal and ventromedial sectors of the AcbC.

Overall, PN harbors a modest amount of mesoaccumbens neurons (about 8%) but this percentage increased to 16% or even 32% when the tracer deposit was confined to the AcbSh and the dorsal AcbC. The greatest percentage of retrogradely labeled neurons in the PN was found when the deposit was placed close to the ac at the mid-rostrocaudal axis of the AcbC, an accumbal territory that was also targeted by many neurons located at the most ventral sector of PBP just dorsal to the PN (see red deposit in **Table 1** and **Figures 3, 5**). In contrast, more ventrally located AcbC deposits near the boundary with AcbSh were more abundantly targeted by neurons located in the dorsal aspects of PBP (**Figure 5**). It is worth noting that retrogradely labeled neurons were virtually absent at lateral aspects of the PBP when the injection sites were located at medial territories of AcbC (**Figures 2, 3**), however, deposits located more laterally within the AcbC resulted in high amounts of labeled neurons in the lateral PBP sector and even some in the SNC (**Figure 4**). Lateral AcbC deposits produced as much as 28 and 34% of the total retrograde labeling in the lateral PBP (**Table 1**). With respect to the AcbSh, our results indicate that small territories of this accumbal division receive inputs from neurons scattered in every VTA subdivision, with high contributions from the PBP and the PN (see yellow tracer deposit in **Figure 1** and **Table 1**; see Supplementary Figure S1). Within the PBP, the retrogradely labeled neurons from the AcbSh deposit were especially abundant in the dorsal aspect of the medial PBP (**Figure 5**). The rVTA contributed to the innervation of every accumbal territory explored in the present study, although its contribution to the total averaged about 7%.

With respect to the neurochemical (TH+ or TH-) phenotype of the retrogradely labeled neurons, our data indicate that all the accumbal zones analyzed here were innervated by both TH+ and TH- VTA neurons, and the proportion of the two neurochemical profiles varied depending on the location of the deposits within the Acb and the VTA subdivisions (**Table 1**). We found that a single VTA subdivision contributes both TH+ and TH- neurons to accumbal innervation. Overall, the proportions of dopaminergic (i.e., TH+) and non-dopaminergic (TH-) neurons innervating a given accumbal zone was fairly close to 50% and this was roughly conserved in the projections that arose from a single VTA subdivision (**Table 1**). A clear exception to this proportion was found in the injection site located at the caudal and ventromedial corner of the AcbC (orange deposit in **Figure 1**) in which 27 of the 36 retrogradely labeled neurons (i.e., 75%) were non-dopaminergic (**Table 1**). The fact that many CLi neurons projecting to this zone were TH- clearly contributed to this high proportion of non-dopaminergic projections.

Finally, all the injections in the Acb, with the exception of that coded in red in **Figure 1**, provided a small amount of retrogradely labeled cells in the contralateral VTA, but only at PBP and CLi subdivisions (**Figures 2, 4**; see Supplementary Figure S1). The contralateral mesoaccumbens neurons were more abundant following the AcbSh deposit and were located in the whole mediolateral extent of PBP, whereas injections in AcbC provided fewer contralateral cells and these were restricted to the medial PBP.

### Single-Neuron Tracing

**FIGURE 6:**
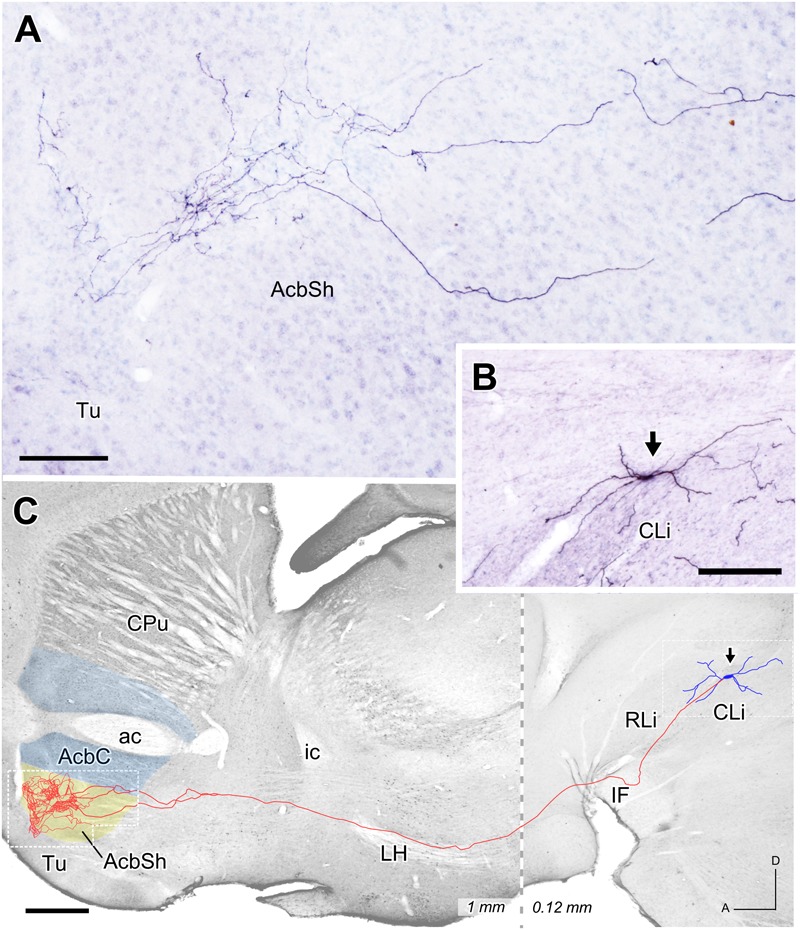
**Mesoaccumbens neuron located at CLi. (A)** Sagittal photomicrograph showing terminal axonal arborizations at the AcbSh after ImageJ processing. **(B)** Sagittal view of the somatodendritic domain (arrow) of the neuron whose complete axon is depicted in **C**. **(C)** Sagittal reconstruction of the CLi neuron superimpose, for reference, over an image of a calbindin-stained sagittal mouse brain section. The dashed line separates the more medial caudal part (0.12 mm) from the more lateral rostral part (1 mm). Bars: **A** = 100 μm; **B** = 200 μm; **C** = 500 μm.

Three mesoaccumbens neurons infected with the Sindbis-pal-eGFP vector were reconstructed in the present study. The confocal analysis of a neuron located dorsally at CLi (**Figure 6**) did not show TH immunoreactivity, indicating that it was presumably non-dopaminergic. This neuron lay quite close to another infected neuron in the CLi but this second neuron was TH+. This TH+ neuron projected to the VP and was not included in the present study. The complete trajectory of the reconstructed TH- neuron is depicted in **Figure 6C**. The axon emerged from the soma and coursed ventrally to traverse the IF, LH and SI without leaving any collateral. Then, as the axon entered the striatum it bifurcated into two major branches that arborized profusely in the rostral and medial aspect of the AcbSh, extending from 0.48 to 1.08 mm from the midline. In the medial extreme of the AcbSh, the preterminal and terminal axonal fibers extended dorsally following the boundary of the nucleus, while in its more lateral aspect they remained ventral, always confined to the AcbSh.

**FIGURE 7:**
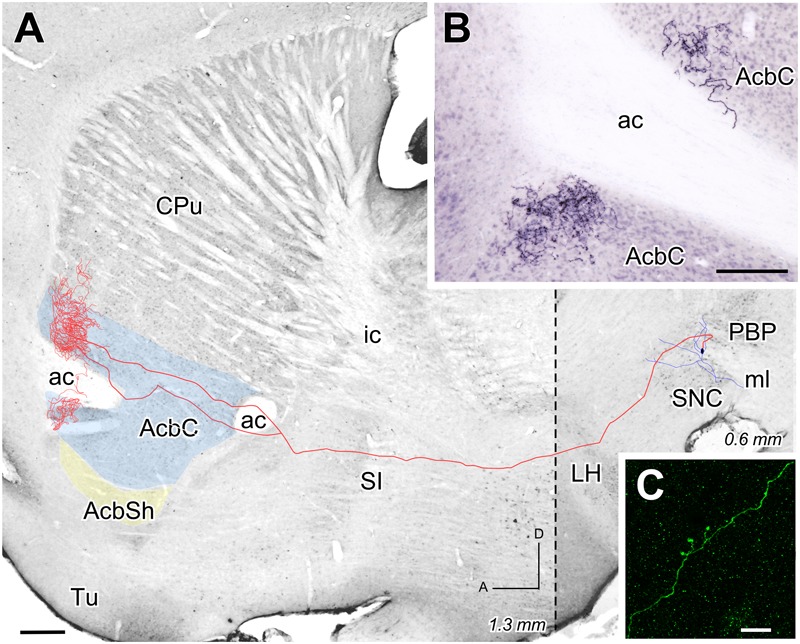
**Mesoaccumbens neuron located at PBP. (A)** Sagittal reconstruction of a neuron located at lateral PBP projecting to superimpose, for reference, over an image of a calbindin-stained sagittal mouse brain section. The dashed line separates (0.6 mm) from the more lateral rostral part (1.3 mm). **(B)** Sagittal photomicrograph showing the terminal field at AcbC on both sides of the ac after Image J processing. **(C)** ImageJ reconstruction from confocal microscopy images of a varicose fiber at AcbC filled with Sindbis-pal-eGFP vector. Bars: **A** = 500 μm; **B** = 100 μm; **C** = 10 μm.

The two other reconstructed neurons belonged to the lateral aspect of PBP and, like the previous reconstructed neuron at CLi, were not seen to express TH immunofluorescence under confocal microscopy. The axon of the PBP neuron shown in **Figure 7** abandoned the PBP dorsally to cross the LH and SI without leaving any collateral. It bifurcated caudally to the ac and provided a dense terminal field that surrounded this commissure dorsally and ventrally (**Figures 7A,B**), and also covered the rostral pole and the rostrodorsal AcbC at its lateral half (1.08 to 1.44 mm from midline). The morphology of a single varicose fiber from this terminal field can be seen in **Figure 7C**, which shows the excellent Golgi-like labeling of axons provided by the Sindbis-pal-eGFP vector even at their more distal arborization. The Acb was also the main target of the other PBP neuron, whose axon, after traveling through LH and SI without giving any collateral, provided a large terminal field at the lateral aspect of AcbC after emitting a collateral that arborized at the adjacent lateral stripe of the striatum.

## Discussion

This work explores the wiring of the mesoaccumbens pathway in mice, by focusing on the contributions of the various VTA subdivisions to the AcbC and AcbSh innervation and on the dopaminergic phenotype, as determined by TH immunoreactivity, of these projections. Small injections of the retrograde tracers FB and FG were placed in different accumbal territories and the distribution of the retrogradely labeled neurons in the various VTA subdivisions was analyzed. Our results demonstrate for the first time that small territories of the AcbC and AcbSh are simultaneously innervated by many VTA subdivisions, which send dopaminergic and non-dopaminergic projections. What is more, a single VTA subdivision contains TH+ and TH- neurons that project into the same accumbal territory. The terminal axonal arborization of VTA neurons is only directed to either the core or the shell of the Acb, but not both, as shown here by a limited sample analyzed by single cell transfection with the Sindbis-pal-eGFP vector.

### Methodological Considerations

The present study assesses the dopaminergic nature of retrogradely labeled VTA mesoaccumbens neurons by simultaneously analyzing their immunoreactivity against TH, which is a reliable marker for dopaminergic neurons in the ventral mesencephalon (Fu et al., 2012). Similar retrograde tracing-immunolabeling methods have been applied to determine the dopaminergic phenotype in previous studies of VTA projections (Sawchenko and Swanson, 1981; Swanson, 1982; Tan et al., 1999; Hasue and Shammah-Lagnado, 2002). We applied state-of the-art double labeling detection on 1 μm-thick confocal optical slices to ascertain the co-localization of retrograde labeling and TH immunofluorescence in VTA cells. Only cell body profiles that were clearly delineated by both labeling techniques were counted. Nevertheless, the possibility remains that TH expression was too low to be detected in some dopaminergic neurons given the inherent limitations of immunolabeling techniques. We observed that VTA innervates the Acb with dopaminergic and non-dopaminergic cells that are intermingled in every VTA subdivision. The existence of substantial Acb innervation from non-dopaminergic VTA neurons is consistent with previous studies in rodents (Swanson, 1982; Tan et al., 1999; Hasue and Shammah-Lagnado, 2002; Ikemoto, 2007).

We did not observe TH immunolabeling in the very limited sample of Sindbis-pal-eGFP vector-labeled neurons whose axons we reconstructed. However, since the Sindbis promoter may interrupt normal cell protein synthesis, a lack of TH labeling does not mean the cell is necessarily non-dopaminergic (Ketola et al., 2005; Aransay et al., 2015). When this vector is used, proteins with a rapid turnover rate, like TH (Tank et al., 1986), could be undetectable 48 h after Sindbis infection.

### Distribution of Retrogradely Labeled Neurons in Each VTA Subdivision

Our results have shown that the VTA subdivision that harbors the highest percentage of retrogradely labeled mesoaccumbens neurons is PBP, specifically its medial territory, a finding that concurs with previous estimations in which PBP was identified as the main input to the medial half of both Acb subdivisions (Yamaguchi et al., 2011). The VTA subdivisions PN and rVTA also contributed to the innervation of all the Acb territories explored in this study although they contained fewer retrogradely labeled neurons than PBP. In the case of the rVTA, its contribution to the total amount of retrogradely labeled mesoaccumbens neurons averaged about 7%, which is somewhat higher than the 2% reported by Yamaguchi et al. (2011) in this VTA subdivision. Regarding the midline VTA subdivisions, the CLi innervates many territories of the AcbC and AcbSh but projects mainly toward the medial AcbC. In contrast, most projections from IF are directly toward specific accumbal territories, such as the caudal and ventromedial AcbC sector, as we have shown in the present study, and the dorsomedial territory of the AcbSh, as reported previously (Thompson and Swanson, 2010). Retrogradely labeled neurons were very scarce in the RLi in all cases analyzed here, a finding that agrees with earlier anterograde studies that showed that the projections from this VTA subdivision largely spare the AcbC providing a sparse innervation in the rostral pole and lateral AcbSh (Del Fava et al., 2007). Only a few contralateral mesoaccumbens neurons were observed in the present study, as previously reported in the rat (Swanson, 1982; Phillipson and Griffiths, 1985). A small contralateral component of the mesoaccumbens pathway has also been described in other similar systems, including the nigrostriatal pathway (Fass and Butcher, 1981; Gerfen et al., 1982).

Dopaminergic and non-dopaminergic retrogradely labeled mesoaccumbens cells were intermingled across the extent of the whole VTA in nearly equal proportions. This parity strongly contrast with a previous estimate made in rats in which dopaminergic cells represented about 80–95% of the total (Swanson, 1982; Tan et al., 1999; Hasue and Shammah-Lagnado, 2002; Ikemoto, 2007), and this difference may be due to species differences. On the other hand, the earlier studies all focused on AcbSh retrograde labeling while the present work mainly analyzes the AcbC, so a distinct pattern of neurochemical innervation from VTA at both Acb subdivisions might explain the reported differences in this dopaminergic innervation.

### Topographic Projections from VTA to Acb

Our results indicate that the medial aspect of the AcbC receives abundant projections from the midline VTA subdivisions IF, CLi and RLi, whereas more laterally in AcbC the percentages of retrogradely labeled mesoaccumbens neurons increased at the medial aspect of PBP. Furthermore, more lateral sectors of the Acb were targeted by neurons located in more lateral aspects of the PBP and eventually in the SNC. This pattern of projections reflects a mediolateral gradient in which medial aspects of the AcbC are more densely innervated by midline nuclei and medial aspects of the PBP, whereas lateral aspects of the AcbC are targeted by neurons located more laterally within PBP and even SNC. This general pattern of VTA projections to the Acb observed in the mouse brain is consistent with previous data in the rat (Phillipson and Griffiths, 1985; Brog et al., 1993) and in the mouse AcbSh (Lammel et al., 2011). A previous study showed that AcbC innervation originated from lateral sectors of VTA and SNC, but not from medial VTA aspects (Ikemoto, 2007), a finding in contrast with our results. This discrepancy could be explained by the small size of our injections involving selectively medial or lateral parts of the AcbC lying ventral to the ac, while the deposits in the previous study involved the dorsolateral AcbC and the rostral pole (see Figure 18 from Ikemoto, 2007), not investigated in the present study. Another report using a large injection of the retrograde tracer FG in the medial AcbC beneath the ac described retrogradely labeled neurons at PBP, PN and midline nuclei (Tan et al., 1999), a pattern that resembles medial AcbSh labeling (Ikemoto, 2007). Along the same lines, IF has been reported to specifically innervate the dorsomedial AcbSh (Ikemoto, 2007; Thompson and Swanson, 2010) and our study has demonstrated that this midline nucleus innervates mostly medial sectors of the AcbC. Altogether these findings support the idea that the mediolateral gradient in the mesoaccumbens projections applies not only to the AcbSh, as widely accepted, but also to the AcbC, at least in the mouse.

Previous studies have pointed out an inverse dorsoventral gradient as a characteristic of the mesostriatal projections from VTA (Aransay et al., 2015) and specifically of the meso-AcbSh pathway (Brog et al., 1993). The results of the present study indicate that this gradient could also be preserved in the meso-AcbC pathway. The existence of a rostrocaudal gradient remains to be confirmed. Early studies reported that the entire rostrocaudal VTA projected to the Acb (Phillipson and Griffiths, 1985), and later studies described projections from the caudomedial VTA, for instance, selectively targeting the medial AcbSh (Ikemoto, 2007). Here, we observed that caudal Acb injections produced fewer retrogradely labeled neurons at rVTA than rostral ones. Mesoaccumbens neurons are distributed through the entire extent of the VTA, intermingled with neurons innervating other targets, in contrast to the projections from Acb to VTA that arise from small and very dense patches of neurons (Watabe-Uchida et al., 2012).

### Arborization Pattern of Mesoaccumbens Axons

Our three individually reconstructed cells – one in CLi and two in PBP- project to rostral areas of the Acb, which we did not explore with the retrograde tracer injections. Despite being located in different VTA subdivisions, the terminal axonal arborization of the neurons is similarly profuse and restricted to either the core or the shell of Acb. Another common feature is the straight, unbranched trajectory of these axons toward the Acb. This feature is in contrast with an earlier study using the same Sindbis-pal-eGFP vector to mark projection neurons in the same VTA subdivisions (Aransay et al., 2015). That study showed abundant axonal branching that innervated multiple basal forebrain, striatal and cortical structures. The lack of axonal branching observed in the present study is not likely to be due to a technical limitation of the vector to fill all axonal branches. The Sindbis-pal-GFP vector has been demonstrated to provide optimal labeling of axonal branches and their terminal arborizations (Furuta et al., 2001; Matsuda et al., 2009; Fujiyama et al., 2011; Aransay et al., 2015). Profuse focal terminal arborization and absence of branches have also been observed in mesostriatal projection neurons labeled with the Sindbis-pal-eGFP vector (Matsuda et al., 2009; Aransay et al., 2015). Therefore, despite the limited number of reconstructed cells, our observations suggest the existence of a population of mesencephalic neurons in the VTA and SNC that only targets the dorsal or ventral striatum. These neurons typically have axons that arborize very profusely and focally in the striatum and without sending branches to other structures.

The CLi subdivision had not been previously explored with single-cell labeling methods. The present findings indicate that CLi mesoaccumbens neurons are similar to those of the PBP and the rVTA (Aransay et al., 2015). It is noteworthy that the CLi neurons project to the medial half of AcbSh while the lateral PBP neurons project to the lateral half of AcbC, supporting the mediolateral gradient noted above. Furthermore, the profuse terminal innervation at the Acb provided by some CLi, PBP and rVTA neurons (present study; Aransay et al., 2015) suggests a powerful and selective control of VTA over the Acb. This could represent the anatomical substrate for the massive response of a high number of Acb spiny neurons after stimulation of very few VTA neurons releasing both dopamine and glutamate (Stuber et al., 2010; Yamaguchi et al., 2011). Finally, our findings complement those described by Beier et al. (2015) in mice, who reported two types of mesoaccumbens neurons (i.e., medial and lateral Acb projecting dopaminergic cells) depending on their pattern of collateralization in other striatal and extrastriatal targets, but did not report the existence of neurons that only projected to the Acb.

## Conclusion

This is the first anatomical study of the mouse mesoaccumbens pathway to analyze the contribution of each VTA subdivision to the innervation of the core and shell Acb subdivisions. Our results indicate that small territories of the AcbC and AcbSh are targeted by many VTA subdivisions, which send dopaminergic and non-dopaminergic projections. Furthermore, a single VTA subdivision contains TH+ and TH- neurons that project into the same accumbal territory.

We did not detect any VTA subdivision displaying a clear topography in its projections toward the Acb, with the exception of the caudal and ventromedial AcbC territory where CLi stands as a major source of projections from the ventral mesencephalon. Overall, the medial domain of PBP is the major source of AcbC afferents from the VTA. The AcbC innervation from VTA follows a mediolateral gradient, as occurs in the AcbSh and other ventral forebrain structures. An inverse dorsoventral gradient, comparable to the one observed in the dorsal striatum or the AcbSh, is also present at AcbC.

Contrasting with observations of other mesoaccumbens neurons in our previous study (Aransay et al., 2015), the limited sample of single-cell labeling descriptions presented here reveal a striking target selectivity and absence of axon collaterals in VTA neurons. We find it particularly remarkable that these axons arborize profusely in terminal fields that do not cross the limit between the two Acb subdivisions. The neuronal wiring of the mesoaccumbens pathway thus seems to be organized in a highly parallel or modular fashion.

## Author Contributions

CR-L: Acquisition of the data related to retrograde fluorescence tracers and single neurons. Analysis, interpretation and drafting of the whole manuscript. FC: Revising the manuscript for important intellectual content. LP: Analysis, interpretation of data and revising the whole manuscript.

## Conflict of Interest Statement

The authors declare that the research was conducted in the absence of any commercial or financial relationships that could be construed as a potential conflict of interest.

## Abbreviations

ac: anterior commissure
Acb: nucleus accumbens
AcbC: nucleus accumbens, core
AcbSh: nucleus accumbens, shell
BSA: bovine serum albumin
BST: bed nucleus of the stria terminalis
C: caudal coordinate
CLi: caudal linear nucleus
cp: cerebral peduncle
CPu: caudate-putamen
D: dorsal coordinate
FB: fast blue
FG: fluorogold
fr: fasciculus retroreflexus
GFP: green fluorescent protein
ic: internal capsule
IF: interfascicular nucleus
IP: interpeduncular nucleus
IPAC: interstitial nucleus of the posterior limb of the anterior commissure
L: lateral coordinate
LH: lateral hypothalamus
LV: lateral ventricle
M: medial coordinate
ml: medial lemniscus
mlf: medial longitudinal fasciculus
NGS: normal goat serum
PB: phosphate buffer
PBP: parabrachial pigmented nucleus
PBS: phosphate buffer saline
PN: paranigral nucleus
RLi: rostral linear nucleus
RRF: retrorubral field
rVTA: rostral part of the ventral tegmental area
SI: substantia innominata
SNC: substantia nigra, pars compacta
SNR: substantia nigra, pars reticulata
TH: tyrosine hydroxylase
Tu: olfactory tubercle
VP: ventral pallidum
VTA: ventral tegmental area
vtgx: ventral tegmental decussation
VTT: ventral tegmental tail
xscp: decussation of the superior cerebellar peduncle
