# Supplementary material for: The Mesoaccumbens Pathway: A Retrograde Labeling and Single-Cell Axon Tracing Analysis in the Mouse
Source: Front Neuroanat. 2017 Mar 27;11:25. doi: 10.3389/fnana.2017.00025 (PMC5367261; doi:10.3389/fnana.2017.00025)
Supplement: Supplementary file 1 [file Table_1.DOCX]

**Table 1. Animal cases analyzed in the present study**

| **Mice** | **Case*** | **Hemisphere** | **Tracer/Location** | **Number of labeled VTA neurons** |
| --- | --- | --- | --- | --- |
| 1 | Orange | Left | FB/AcbC | 36 |
|  | Pink | Right | FG/AcbC+Sh | 45 |
| 2 | Red | Right | FB/AcbC | 31 |
|  | Blue | Left | FG/AcbC | 38 |
| 3 | Yellow | Right | FB/AcbSh | 217 |
|  | Green | Left | FB/AcbC+Sh | 129 |
| 4 |  | Right | Sindbis-pal-eGFP /CLi | 3 |
| 5 |  | Left | Sindbis-pal-eGFP /PBP | 1 |
| 6 |  | Left | Sindbis-pal-eGFP /PBP | 1 |

***** The colors correspond to those indicated in Table 1 and Figures 1 and 5. For abbreviations see list of abbreviations.
