# Supplementary figures and images for: The Mesoaccumbens Pathway: A Retrograde Labeling and Single-Cell Axon Tracing Analysis in the Mouse
Source: Front Neuroanat. 2017 Mar 27;11:25. doi: 10.3389/fnana.2017.00025 (PMC5367261; doi:10.3389/fnana.2017.00025)

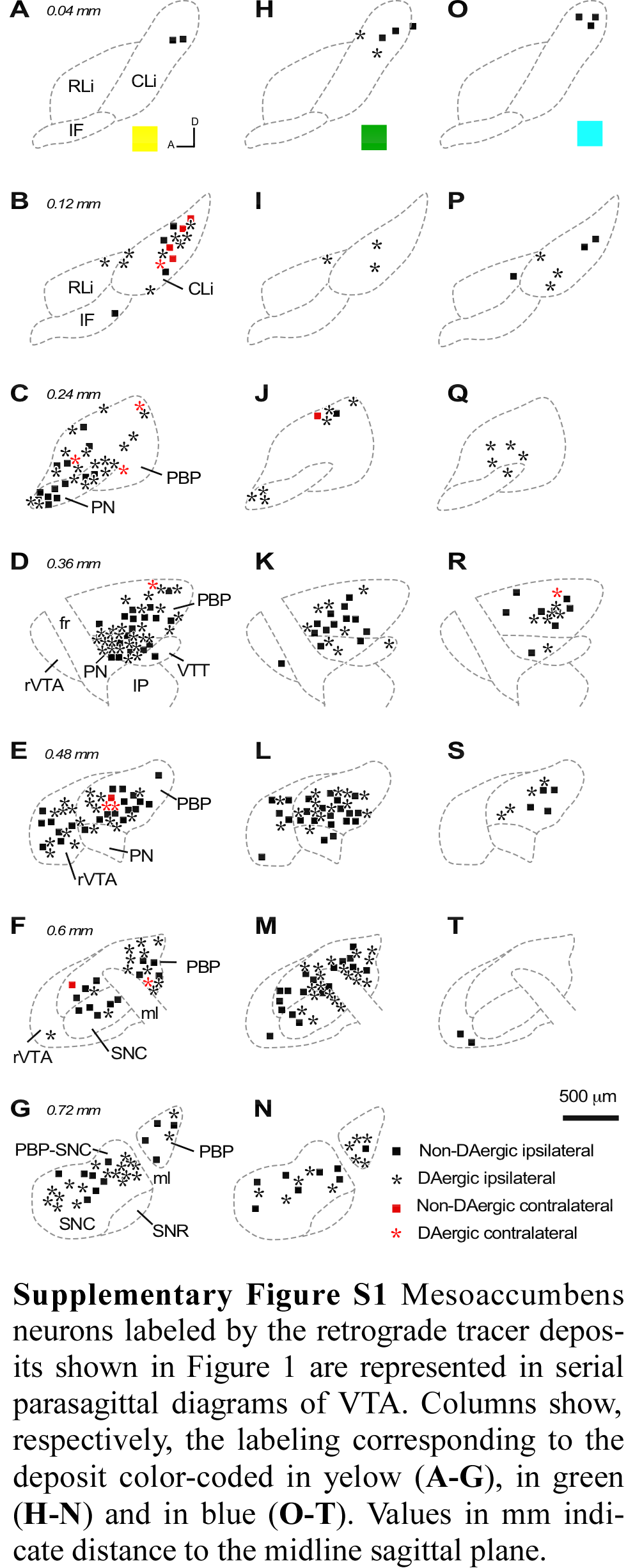

Supplement: Supplementary file 2 [file Image_1.tif]
